# National Epilepsy Month — November 2013

**Published:** 2013-11-01

November is National Epilepsy Awareness Month. Epilepsy is a brain disorder characterized by recurrent seizures; it affects approximately 2.3 million adults in the United States (1).

The CDC Prevention Research Centers’ Managing Epilepsy Well (MEW) Network includes U.S. universities, the Epilepsy Foundation, and other epilepsy groups (2). The MEW Network works to develop and test programs that improve self-management and quality of life for persons with epilepsy.

Several MEW Network programs are available. WebEase is an Internet-based program (https://www.epilepsyfoundation.org/livingwithepilepsy/webease/index.cfm) shown to improve some epilepsy self-management outcomes (3). UPLIFT is an Internet and telephone-based program to treat depression in adults with epilepsy (4), with training for health-care providers available at http://www.sph.emory.edu/ManagingEpilepsyWell/UPLIFT. PEARLS is a collaborative-care depression treatment program for adults with epilepsy (5) with training for health-care providers available at http://www.pearlsprogram.org. Additional information regarding the MEW Network and related resources (such as webinars and podcasts) is available at http://www.cdc.gov/epilepsy and @mewnetwork on Twitter.
